# Unlocking the Past to Protect the Future: Forensic DNA and Historical Specimens Reveal the Origins of Smuggled Yellow‐Headed Parrots

**DOI:** 10.1111/eva.70282

**Published:** 2026-06-14

**Authors:** Brenda R. Ramirez, Devon A. DeRaad, Allison E. Muhlheim, Pat Latas, Brooke Durham, Nikki Buxton, Marquette J. Mutchler, Eliza J. Kirsch, Whitney L. E. Tsai, Alana K. Pizarro, John E. McCormack

**Affiliations:** ^1^ Moore Laboratory of Zoology Occidental College Los Angeles California USA; ^2^ Department of Ecology & Evolutionary Biology UCLA Los Angeles California USA; ^3^ Wild Parrot Coalition Tucson Arizona USA; ^4^ SoCal Parrot Jamul California USA; ^5^ Belize Bird Rescue Belmopan Belize

## Abstract

Wildlife trafficking poses a major threat to biodiversity, particularly for species like parrots that are targeted in the pet trade. The Yellow‐headed Parrot ( *Amazona oratrix* ), an endangered species native to Mexico and Central America, is frequently poached from unknown geographic locations, hindering the identification of smuggling sources and repatriation of any confiscated individuals. Here, we used population genomic tools to determine the source of 18 Yellow‐headed Parrots confiscated as nestlings during a 2021 U.S.–Mexico border crossing into California. To build a genomic reference map, we extracted and sequenced DNA derived from the toe pads of historical museum specimens from across the range of three out of the four described *A. oratrix* subspecies. Genomic data revealed that the three sequenced subspecies correspond to unique ancestry groups and revealed a substantial genetic divide between Pacific and Atlantic coastal populations of *A. o. oratrix*. The smuggled parrots and parrots from the introduced population in southern California both consistently clustered with eastern populations of *A. o. oratrix* in genomic analyses. Concordantly, the predicted origin of these smuggled birds was eastern Mexico, based on the genomic reference map derived from historical samples. Kinship analysis reveals multiple cases of sibling‐level relationships among the smuggled birds, suggesting that wild *A. oratrix* nests continue to be raided for the pet trade despite the species' endangered status. Overall, this study illustrates the power of using museum genomics to trace wildlife trafficking routes, inform conservation policy, and improve taxonomic resolution in species of conservation concern.

## 1 Introduction

Wildlife trafficking is a multi‐billion‐dollar industry that contributes to global species declines and extinctions (Cardoso et al. 2021; Wyatt et al. 2020; Tinsman et al. 2025). Enforcement strategies have often struggled to trace the origins and smuggling routes of trafficked species, but advances in DNA sequencing technology provide powerful forensic tools for combating illegal trade (Butler 2023; Kanthaswamy 2024). Genetic methods allow for species identification and the reconstruction of population structure to determine geographic origins of confiscated individuals (Ogden and Linacre 2015; Wasser et al. 2008; Heighton et al. 2026). These approaches have proven useful for enforcing conservation laws, but the methods are still developing.

Parrots (family Psittacidae) are particularly vulnerable to trafficking due to their popularity in the pet trade and international restrictions on the commercial trade of wild‐caught individuals under CITES, as well as national laws such as the 1992 U.S. Wild Bird Conservation Act (WBCA), although captive‐bred parrots may still be legally sold in many jurisdictions. More than 30% of the approximately 400 parrot species are at risk of extinction, with illegal poaching playing a significant role in their declines (Wright et al. 2001). Latin America is a hotspot for parrot trafficking, with many species captured in the wild and smuggled across international borders, particularly into the United States (Pires 2012).

**FIGURE 1:**
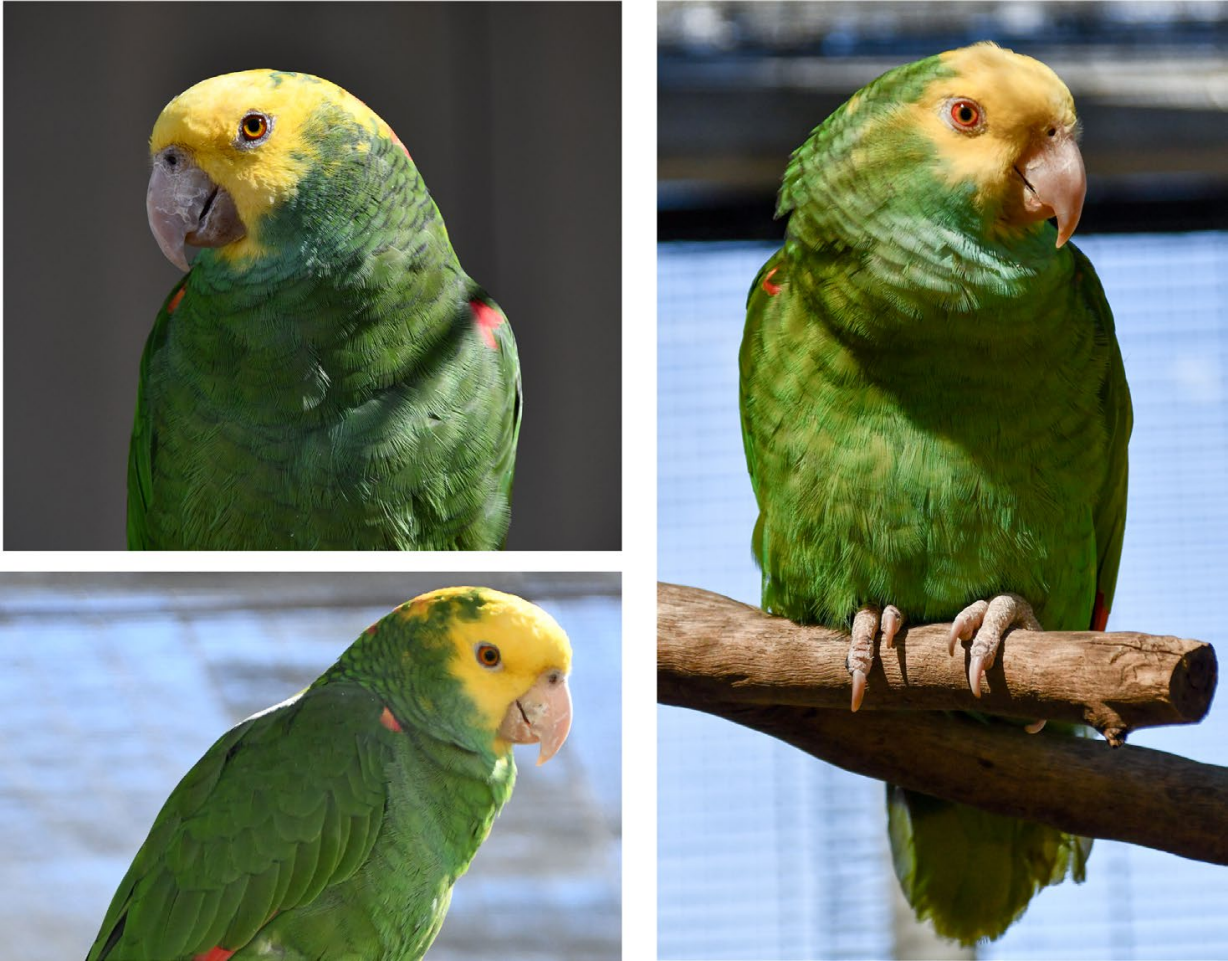
Photographs displaying three of the 18 *A. oratrix* that were confiscated and subsequently raised at SoCal Parrot, a licensed wildlife rehabilitation facility in Southern California. Photo credit Dez Brooks.

The Yellow‐headed Parrot ( *Amazona oratrix* ), an endangered species native to Mexico and Central America (Figure 1), is frequently targeted due to its striking coloration and ability to mimic human speech (BirdLife International 2020; Tella and Hiraldo 2014). This species has suffered a 68% decrease in its population size over the last 10 years due to habitat loss and poaching activities (BirdLife International 2020). It has not been legal to import Yellow‐headed Parrots into the US since 1993, and a 2007 ban on the wild capture and commercial breeding of parrots in Mexico has led to a decline in illegal trade; yet this species continues to be one of the most seized by customs enforcement (Saldaña et al. 2007; Cantú Guzmán et al. 2022). Identifying the geographic source of confiscated Yellow‐headed Parrots is therefore critical for conservation, as this information can inform targeted anti‐poaching strategies and guide responsible repatriation and rehabilitation efforts. Yet, the geographic origin of confiscated individuals is rarely known from seizure context alone, as trafficking networks often obscure provenance through undocumented capture, translocation across multiple regions, and interception far from source populations, creating a critical information gap that genomic tools are uniquely positioned to address.

**FIGURE 2:**
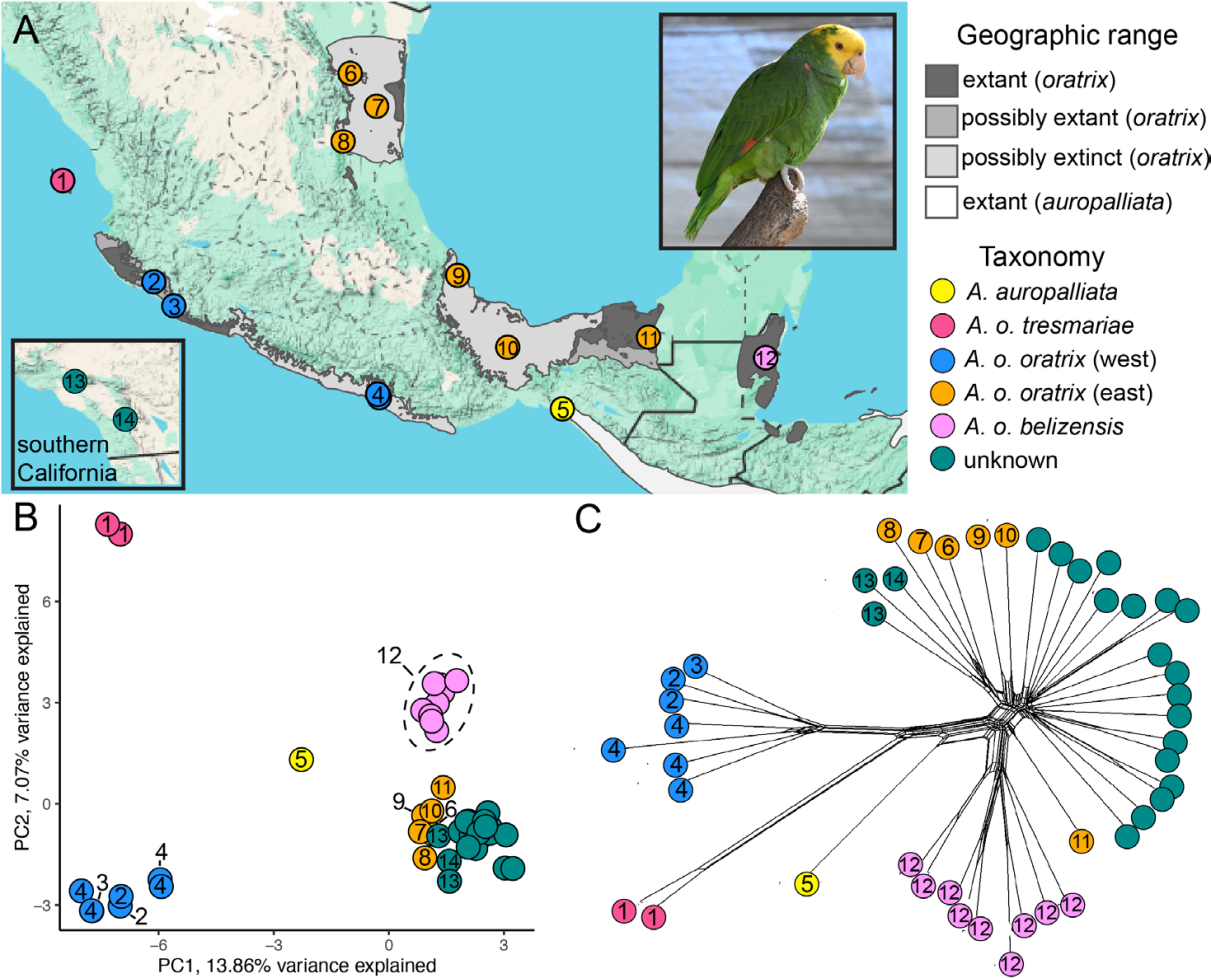
Genetic clustering of smuggled parrots compared to samples from the native range of *A. oratrix* and *A. auropalliata* . (A) Sampling localities from the native ranges of *A. oratrix* and *A. auropalliata* used to construct the genomic map. Color scheme for the distribution of *A. oratrix* follows IUCN designations. Note that the range of *A. auropalliata* extends into central America, beyond the boundary of this map. Inset photo shows a confiscated Yellow‐headed Parrot, photo credit Dez Brooks. (B) Genomic PCA of all individuals that passed filtering (2274 SNPs), color‐coded and labeled according to sampling locality in the map above. Note that smuggled parrot samples have no designated sampling locality. (C) An unrooted phylogenetic network displays genetic distance between all sequenced individuals using the same SNP dataset.

In this study, we used genomic tools to trace the origins of 18 Yellow‐headed Parrots confiscated at a U.S.‐Mexico border crossing in California in 2021. We generated range‐wide genomic data from the species' native range (Figure 2A) using DNA extracted primarily from historical museum specimens from known sampling localities across the species range, allowing us to compare this genomic map with DNA sequenced from these trafficked parrots. By integrating historical museum specimens with modern genomic methods, we provide new insights into the illegal parrot trade and demonstrate a role for natural history collections in aiding contemporary efforts to combat wildlife trafficking.

## 2 Methods

### 2.1 Sampling and DNA Extraction

We sampled 53 Yellow‐headed Parrots ( *Amazona oratrix* ) representing the species' native range (Table S1), using historical and modern sources. We also sequenced a single Yellow‐naped Amazon *A. auropalliata* , which is a closely related and geographically proximate species that prior studies have suggested is paraphyletic with *A. oratrix* (Russello and Amato 2004). Samples from Belize were all chicks originating from Protected Areas (e.g., Payne's Creek National Park, Swasey/Bladen/Deep River Forest Reserves) that were rescued, hand‐raised, and finally released back to their native areas in 2022. DNA was extracted from 10 blood card samples from Belize (*A. o. belizensis*), a single toe pad from a museum specimen from Honduras (*A.o. hondurensis*), and 22 toe pads from museum specimens collected from throughout Mexico, including one *A. auropalliata* individual from Chiapas. Of the 21 Mexican *A. o. oratrix* toe pads 11 were from eastern Mexico, eight were from western Mexico, and two were from the Islas Marías (*A. o. tresmariae*) off the coast of Nayarit. We also included three tissue samples from the naturalized population of Yellow‐headed Parrots located in Southern California. Finally, we included blood samples drawn from 18 Yellow‐headed Parrots of unknown origin housed at SoCal Parrot, a licensed wildlife rehabilitation facility in Southern California. These parrots were confiscated as nestlings in 2021 at a U.S.‐Mexico border crossing in California as they were being illegally transported in car door panels. Digital photos of some of these parrots were taken by SoCal Parrot Operations Director Dez Brooks in February 2026 (Figure 1). DNA was purified from blood and tissue samples using the silica‐based DNeasy Blood and Tissue Kit (QIAGEN). All museum toe pad samples were extracted following a phenol:chloroform:isoamyl alcohol protocol and handled with appropriate contamination controls for historical DNA, including using dedicated equipment in a clean room and negative controls (McCormack et al. 2016; Tsai et al. 2020).

### 2.2 UCE Sequence Capture and SNP Calling

To generate thousands of genome‐wide, unlinked markers for fine‐scale population assignment, we targeted single‐nucleotide polymorphisms (SNPs) derived from ultraconserved elements, or UCEs (Faircloth et al. 2012; McCormack et al. 2012). Library preparation followed standard protocols with a KAPA Hyper prep kit. Indexed DNA samples were enriched for 5060 tetrapod UCE loci using synthetic RNA probes (MYbaits_Tetrapods‐UCE‐5K kit, Arbor Biosciences). Samples were pooled in batches of eight, with historical and modern DNA processed in separate pools because DNA libraries with smaller insert sizes (e.g., those obtained from degraded DNA derived from historical museum specimens) will preferentially bind to the flow cell, resulting in uneven sequencing coverage. Following enrichment, pools were quantified via qPCR and sequenced using an Illumina HiSeq X platform with 100 bp paired‐end reads.

Sequencing reads were demultiplexed using bcl2fastq and delivered in fastq format. We then performed adapter trimming and quality filtering using Fastp v0.22.0 (Chen et al. 2018). Specifically, we removed the first five base pairs from each raw read and cut each read off if the sequence data fell below an average phred‐scaled quality score of 30 within a five base pair sliding window moving from the front to the back of each read. We aligned these trimmed reads to a high‐quality reference genome of the congener *Amazona ochrocephala* (GenBank: GCA_039720435.1; Rhie et al. 2021), using BWA v0.7.17. Picard Tools v2.18.29 was used to assign read group information, mark PCR duplicates, and fix mate pair inconsistencies. We used the Genome Analysis Toolkit, v3.8.1 (McKenna et al. 2010) to realign reads, call genotypes, and output all variant sites in variant call format (vcf). This generated an unfiltered SNP dataset containing 54 samples, 736,148 SNPs, and 92.01% overall missing data.

### 2.3 SNP Filtering

We then used the R packages SNPfiltR v1.0.2 (DeRaad 2022) and vcfR v1.14.0 (Knaus and Grünwald 2017) to filter this SNP dataset based on various quality metrics, following the general approach from DeRaad et al. (2023) for deriving high quality SNPs from UCEs sequenced from historical samples. In brief, we retained only bi‐allelic SNPs, filtered for allele balance (i.e., removed heterozygous genotypes where allele balance was < 0.25 or > 0.75), removed genotypes supported by < 3 reads, and removed SNPs with outlier depth (here, mean genotype depth > 100), which are likely to represent paralogous loci. We then removed SNPs that became invariant following the genotype filters described above and performed preliminary clustering analyses.

Based on these preliminary analyses, we removed a single toe pad derived sample (ao_MLZ_32244; Table S1) that clustered outside of all other congeneric samples, suggesting that the DNA sequence data generated for this sample resulted from a contaminated or mislabeled DNA extraction. We also removed seven samples (six historical toe pad derived plus one blood sample; Table S1) that were missing genotypes for > 80% of remaining SNPs, that is, did not generate enough sequence data to be used in downstream analyses. This resulted in the removal of the only representative of the subspecies *A. o. hondurensis*. While characterizing this small and disjunct subspecies is important to understanding the full taxonomy of the species, it represents ~1% of the overall range, and the limited field observations suggest it is unlikely to be a major source population for smuggled birds (Britt 2017). Additionally, recent photos of the smuggled birds as adults (Figure 1) rule out a Honduras source population based on yellow plumage patterning on the head, which in Honduras birds does not surround the eye (Lousada and Howell 1996).

Subsequently, we found that requiring SNPs to be 80% complete, that is, missing < 20% of called genotypes, maximized the tradeoff between number of retained SNPs and overall missing data proportion. Finally, we removed singleton SNPs, i.e., SNPs with a minor allele count (MAC) < 2. This resulted in a filtered SNP dataset of 46 samples, 2274 SNPs, and 7.75% overall missing data (individual sample missingness ranges from 0%–53.7%; Table S1). We also performed linkage filtering, removing all SNPs closer than 1 Kbp, to generate a filtered, unlinked SNP dataset containing 46 samples, 1598 SNPs, and 8.01% overall missing data. This unlinked SNP dataset showed qualitatively similar sample clustering patterns as the full SNP dataset (Figure S2), albeit with less power to discriminate between subspecies, therefore the full SNP dataset was used for all analyses presented in the main text of the manuscript.

### 2.4 Assessing Sample Relatedness

To explore patterns of genetic relatedness in our SNP dataset, we performed a principal components analysis (PCA) using the function glPca() from the R package adegenet v2.1.11 (Jombart 2008). We used our filtered SNP dataset (46 individuals, 2274 SNPs) as input and visualized sample clustering across the first two PC axes using ggplot2 (Wickham 2016). To further investigate patterns of fine‐scale structuring, we subset our filtered SNP dataset to only include samples from the *A. o. oratrix* (east) and *A. o. belizensis* clades, plus the samples of unknown taxonomic affinity, and removed any SNPs that became invariant (i.e., MAC < 1). We then used this subset dataset (36 individuals, 1819 SNPs) to generate and visualize PCA clustering following the procedure described above. To assess sample relatedness in a phylogenetic context, we also used the filtered SNP dataset (46 individuals, 2274 SNPs) to create an unrooted, distance‐based phylogenetic network. To do this, we used the R package StAMPP v1.6.3 (Pembleton et al. 2013) to create a pairwise genetic distance (Nei's D; Nei 1972) matrix among all samples and used this matrix as input for SplitsTree v4.19.1 (Huson and Bryant 2006) to construct an unrooted NeighborNet that visualizes the genetic distance between all samples in a single two‐dimensional network.

We then sought to directly quantify genetic distance both between taxonomic groups and between individuals. We began by constructing a pairwise kinship matrix between all sequenced individuals passing filtering protocols. To do this, we ran PLINK v2.0.0 (Chang et al. 2015) on the filtered SNP dataset (46 individuals, 2274 SNPs) using the flag “‐make‐king‐table” to calculate pairwise KING kinship coefficients (Manichaikul et al. 2010). We then visualized the resulting kinship coefficients as a heatmap color‐coded based on the categories designated for this statistic by McMaster et al. (2025), specifically: identical (e.g., monozygotic twin/clone) > 0.35; first‐order (e.g., parent‐offspring, full sibling) > 0.18 and < 0.35; and second‐order (e.g., grandparent‐grandchild, half sibling) > 0.09 and < 0.18.

To quantify genetic distance between clades we assigned all samples into groups according to taxonomy and sampling locality and calculated pairwise F_ST_. Before performing this analysis, we removed the single *A. auropalliata* individual, because F_ST_ calculation relies on allele frequencies and can behave unpredictably when using only a single individual to represent a population. After removing this sample and removing any subsequent invariant sites (i.e., MAC < 1) this left us with a filtered SNP dataset containing 45 individuals and 2248 SNPs. This dataset was used as input for StAMPP to perform the pairwise F_ST_ calculation.

### 2.5 Predicting Geographic Origins

We used the machine learning software Locator v1.2.1 (Battey et al. 2020) to predict the geographic origins of the confiscated Yellow‐headed Parrots. Only individuals with known localities that passed genetic filtering were included in the analysis (Table S1). To avoid bias from overrepresented populations, we only included the two Belizean samples (site 12) with the least amount of missing data and assigned their location to a forested area in Payne's Creek National Park, where these parrots are commonly observed. New VCFs were created to match the sample data associated with each run of Locator predictions (Table S2). Each group of predictions was run with five separate sets of seeds, and model performance was averaged across the runs to assess overall model performance. A representative run was chosen with the closest mean error to the average of all runs.

To assess the predictive ability of our training dataset, we first ran Locator on sample ao_MLZ_48333 from Tamaulipas. For this, we trained and calibrated our model using all known localities from the filtered VCF (excluding any rescued or confiscated parrots) and set sample ao_MLZ_48333 as the unknown. Predictions were generated using 200 bootstrap replicates, an 80% training split to balance model learning and validation, and neural networks with three dense layers to minimize overfitting. This model was run five times with different fixed random seeds to ensure reproducibility and assess model performance. Model performance was evaluated using the mean and median validation errors, the coefficient of determination for latitude and longitude (*R* ^2^), and the accuracy of the predicted sample locations of each model set.

Once we were confident in the predictive ability of our dataset, we then applied the same approach to the 18 confiscated Yellow‐headed Parrots, training the model on known localities (excluding rescued parrots of unknown origin) and generating predictions using the same parameters and the five set seeds. Similarly, we predicted the origin of three Yellow‐headed Parrot samples from Southern California, using the same parameters (excluding confiscated parrots of unknown origin) and five set seeds.

## 3 Results

### 3.1 Assessing Individual Ancestry

Principal components analysis (PCA) of the filtered SNP dataset revealed clear population structure between the three sequenced *Amazona oratrix* subspecies across their native range on both PC1 (13.86% variance explained) and PC2 (7.07% variance explained; Figure 2B). Additionally, we uncovered a clear genetic break between the eastern and western populations of the coastal subspecies, *A. o. oratrix*. The single *A. auropalliata* individual included in this analysis falls in the center of the PCA, suggesting that it is embedded within the diversity of *A. oratrix* . All individuals of unknown ancestry (both the smuggled birds and those sampled from the introduced population in southern California) clustered with *A. o. oratrix* populations sampled from eastern Mexico in the PCA plot.

An unrooted phylogenetic network (Figure 2C) revealed similar patterns, with each subspecies again forming a discrete cluster. The lone *A. auropalliata* sample was again embedded within the overall genetic diversity of *A. oratrix* . Notably, the smuggled birds were recovered in the network between *A. o. oratrix* samples from adjacent localities in southeastern Mexico (localities 9–11). Meanwhile, samples derived from the introduced population in southern California formed a separate cluster with *A. o. oratrix* samples from Tamaulipas and Nuevo León (localities 6–8), in northeastern Mexico.

### 3.2 Predicting the Geographic Origin of Samples With Unknown Taxonomic Affinity

Locator model performance for five trial runs predicting the geographic origin of a single sample of known provenance (sample ao_MLZ_48333; collected from Tamaulipas) correctly identified the sample as coming from the eastern *A. o. oratrix* clade (Table S3). Yet, the mean of the 200 bootstrap predictions was approximately 361 km away from the true origin of the sample (Figure S1), between the northeastern and southeastern populations of *A. o. oratrix*, suggesting limited statistical power of Locator to differentiate these two weakly diverged populations (Figure 2B).

**FIGURE 3:**
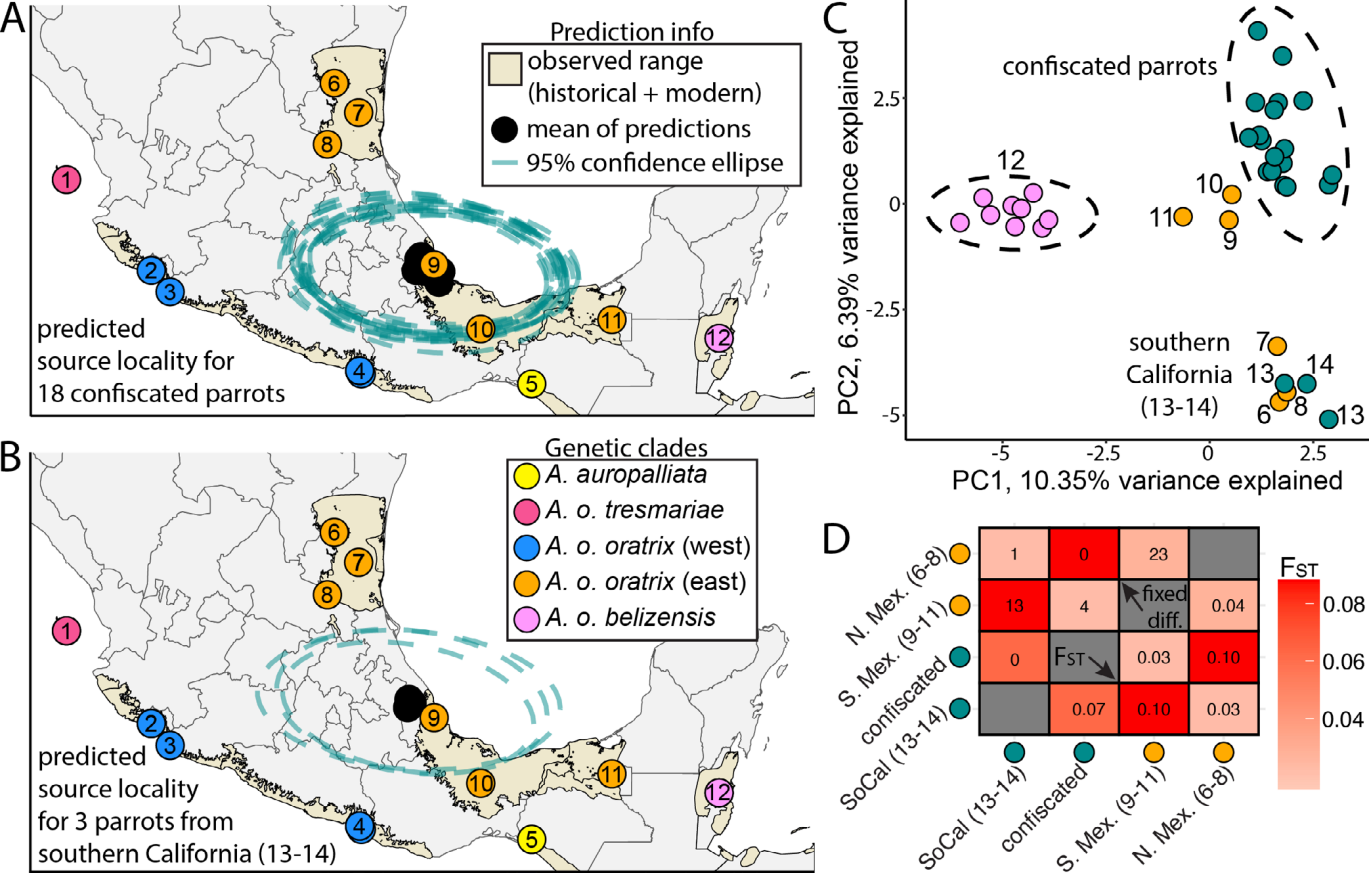
Geographic assignment of confiscated and introduced *Amazona oratrix* samples. (A) Each black dot represents the predicted source locality for a single smuggled *A. oratrix* individual. Each dashed ellipse represents the extent of the 95% confidence interval (based on 200 bootstrap replicates) for the predicted locality of a single individual. (B) Locator predictions of the source locality for three individuals sampled from southern California. (C) Genomic PCA based on 1819 SNPs and including only samples from *A. o. oratrix* (east) and *A. o. belizensis*, plus samples with unknown taxonomic affinity, reveals fine scale genetic differentiation. Ellipses are included to visually group individuals assigned to the same genetic cluster. (D) Pairwise F_ST_ (shown below the diagonal) and pairwise fixed differences (shown above the diagonal) between relevant groups. fixed diff., fixed differences; N. Mex., northeastern Mexico; S. Mex., southeastern Mexico; SoCal, southern California.

For the 18 confiscated parrots, model performance was on par with model performance during our test run using a sample of known provenance (Table S3). The 200 bootstrap replicates for each sample consistently converged on a mean predicted locality in eastern Mexico, centered around the Veracruz and Tabasco populations (Figure 3A). Similarly, the three individuals sampled from southern California were predicted to originate from eastern Mexico, again near Veracruz and Tabasco (Figure 3B). Based on these large confidence intervals, this method does not have statistical power to differentiate whether the confiscated parrots or the Southern California population are derived from northeastern versus southeastern Mexico.

Patterns of fine‐scale clustering among samples were revealed in a PCA including only the *A. oratrix* populations found on the east coast of Mexico and central America (Figure 3C). On PC1, samples of unknown taxonomic affinity are indistinguishable from samples derived from the eastern clade of *A. o. oratrix*. But, on PC2, it becomes clear that samples from southern California cluster most closely with *A. o. oratrix* samples from localities 6–8 in northeastern Mexico, while the confiscated individuals cluster most closely with *A. o. oratrix* populations 9–11 in southeastern Mexico. These patterns of fine‐scale differentiation are also generally supported by pairwise F_ST_ values and fixed differences (Figure 3D) as well as our unrooted phylogenetic network (Figure 2C). For instance, we found a closer relationship between the southern California population and northeastern Mexico (F_ST_ = 0.03; fixed differences = 1) than between southern California and southeastern Mexico (F_ST_ = 0.10; fixed differences = 13). In contrast, we found a closer relationship between the confiscated birds and southeastern Mexico (F_ST_ = 0.03; fixed differences = 4) than between the confiscated birds and northeastern Mexico (F_ST_ = 0.10; fixed differences = 0). These patterns suggest that fine‐scale population differentiation may be possible despite the limited performance of Locator with our data.

### 3.3 Assessing Genetic Relatedness

**FIGURE 4:**
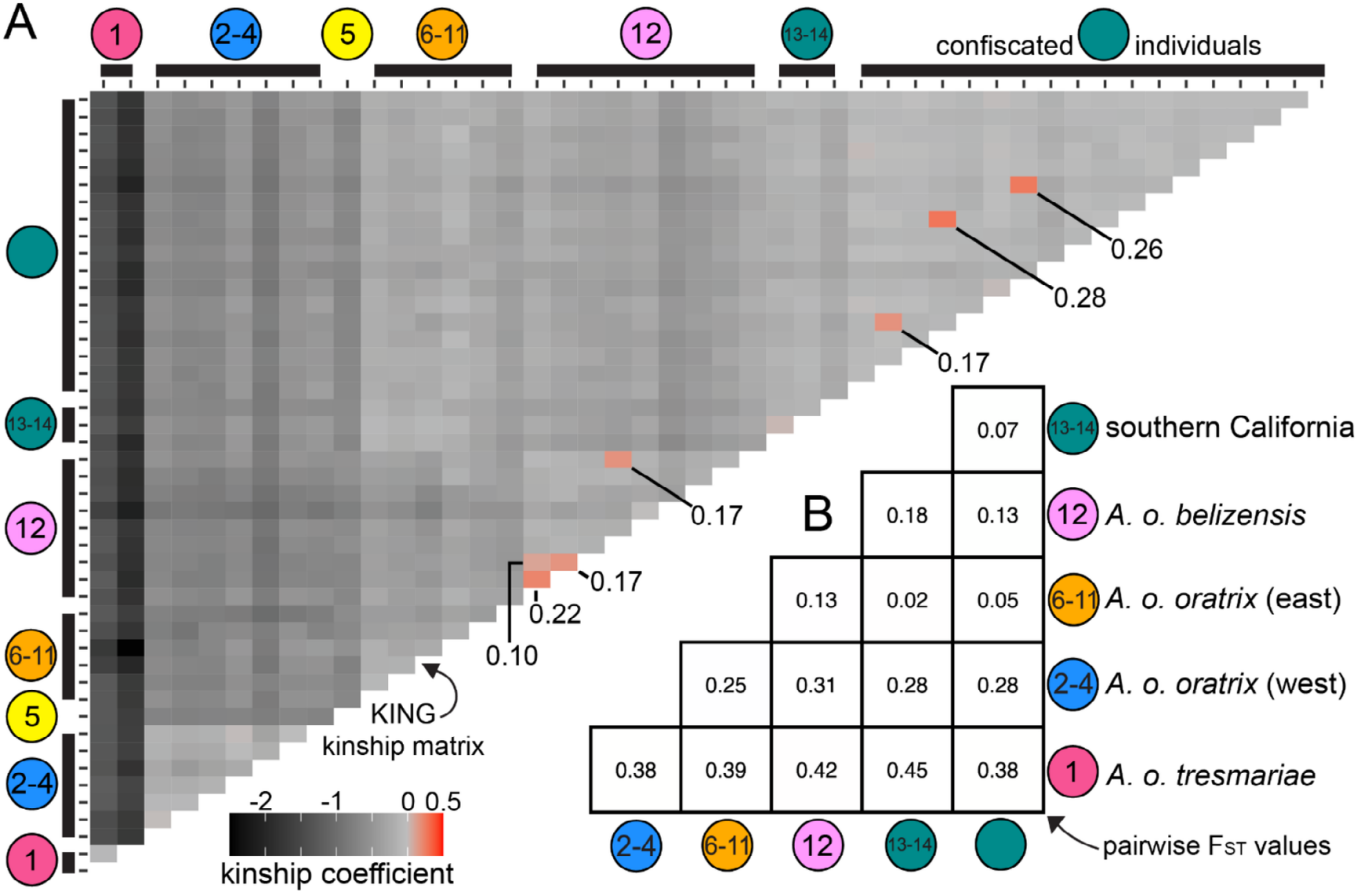
Pairwise kinship estimates and population differentiation (F_ST_). (A) Heatmap showing the pairwise kinship coefficient values between all 46 samples that passed genomic filtering. Pairwise comparisons implying first or second order relatedness (i.e., values > 0.09) are labeled to show the exact kinship coefficient. (B) Pairwise F_ST_ between major taxonomic groups plus populations of interest with unknown taxonomy. Color‐coding and population numbering matches previous figures.

We then identified potential familial relationships by computing a KING kinship matrix (Manichaikul et al. 2010), which is robust to population substructure. The pairwise kinship coefficient between two birds is expected to be zero in the case of unrelated individuals in a randomly mating population. Therefore, a positive kinship coefficient indicates familial relatedness or the presence of inbreeding in the population, while a negative kinship coefficient suggests that the two individuals belong to different subpopulations. Within each subpopulation values generally hovered around zero, suggesting a general pattern of random mating among wild *Amazona* populations (Figure 4A). Notable exceptions included the nine sequenced *A. o. belizensis* birds, which were brought to a wildlife rehabilitation center as a group, among which we identified one first order (kinship coefficient > 0.18 and < 0.35; e.g., parent‐offspring or full sibling) and three second‐order (kinship coefficient > 0.09 and < 0.18; e.g., grandparent‐grandchild or half sibling) instances of kinship. Additionally, among the 18 confiscated parrots, we identified both first order (two cases) and second order kinship (one case). In cross‐population comparisons between wild populations from the species' native range (sites 1–11) values were always negative, corroborating the population structure identified in previous analyses.

Pairwise F_ST_ calculated between populations revealed deep divergence between the Islas Marías endemic subspecies *A. o. tresmariae* and all other lineages (F_ST_ = 0.38–0.45; Figure 4B), as expected based on branch lengths and PCA clustering patterns (Figure 2B,C). Additionally, there is greater relative genetic divergence between *A. o. oratrix* populations on the Pacific (west) and Atlantic (east) coasts of Mexico (F_ST_ = 0.25) than between some subspecies (e.g., *A. o. belizensis* vs. *A. o. oratrix* east, F_ST_ = 0.13), underscoring the inaccuracy of current subspecific taxonomy. Supporting previous analyses, the confiscated parrots demonstrate the lowest relative genetic divergence in comparisons with the *A. o. oratrix* (east) population (F_ST_ = 0.05), as do the parrots from southern California (F_ST_ = 0.02).

## 4 Discussion

### 4.1 Tracing the Geographic Origins of Confiscated Parrots

The genomic reference set for *A. oratrix* , built largely from DNA derived from historical museum specimens, shows significant genomic differentiation between all three sequenced subspecies, plus an additional phylogeographic break between eastern and western populations within *A. o. oratrix*. The 18 Yellow‐headed Parrots confiscated in 2021 at the U.S.‐Mexico border consistently group with the *A. o. oratrix* lineage found in eastern Mexico in all genomic analyses. Further, some of our analyses suggested fine‐scale genetic clustering of these birds with historical museum samples sourced specifically from Veracruz, Tabasco, and Campeche in southeastern Mexico. This area is a known hotspot for illegal poaching (Saldaña et al. 2007) and harbors one of the largest extant populations of Yellow‐headed Parrots in Mexico. Interestingly, while samples from the introduced population in Southern California also consistently cluster with the eastern lineage of *A. o. oratrix*, fine scale clustering patterns suggest that they are most similar to samples from the northeastern states of Tamaulipas and Nuevo León. Ultimately, the introduced population in southern California, which arrived via the pet trade in the early 1960s (Monroe and Howell 1966; Garrett 1997), is likely a mix of ancestry due to the ongoing releases (intentional or accidental) of captive *A. oratrix* in the region.

Our dataset included thousands of SNPs, which provided sufficient resolution to distinguish major lineages within *A. oratrix* , but displayed limited ability to differentiate closely related geographic populations (e.g., northeastern and southeastern Mexico, pairwise F_ST_ = 0.04; Figure 3; Figure S1), an area where future whole‐genome sequencing efforts could offer additional resolution. We also note that our genomic analyses did not include the subspecies *A. o. hondurensis*, although this is unlikely to be the source population for these smuggled birds due to known differences in plumage patterning of the adults. Additionally, our sampling does not include introduced (assumedly via the pet trade) populations of *A. oratrix* from urban areas in Mexico, such as Saltillo, Nuevo León, or Tuxtla Gutiérrez, Chiapas. It is therefore possible that these introduced urban parrot populations in Mexico are derived from the eastern lineage of *A. o. oratrix*, and the confiscated parrots we sequenced were collected from these urban areas. However, what is known about the illegal parrot trade in Mexico suggests that it is more likely that these smuggled birds were taken directly from the cavity nests of wild *A. oratrix* individuals (Saldaña et al. 2007). This conclusion is further supported by our kinship analysis (Figure 4A), which identified multiple sibling‐level relationships among the confiscated birds, implying that these parrot chicks were collected as eggs or nestlings and subsequently moved as sibling groups.

### 4.2 Leveraging Historical Specimens to Build Genomic Reference Maps

This study highlights the growing potential of historical museum specimens to serve as the foundation for genomic reference maps to understand species identification and smuggling routes for threatened and endangered species (Coghlan et al. 2012; Ewart et al. 2018). For many such taxa, legal and logistical constraints make the collection of modern genetic samples difficult, if not impossible. As a result, building comprehensive reference datasets based solely on contemporary material is often impractical and will not include historical areas of residence where species are now extirpated.

Advances in historical DNA methodologies, coupled with next‐generation sequencing technologies, have transformed the world's network of natural history collections into invaluable genomic resources (McCormack et al. 2017, 2016). In this case, specimens from six museum collections—spanning more than a century of collecting effort—provided the necessary geographic coverage to construct a map of genomic relatedness in *Amazona oratrix*, which allowed us to confidently identify the subspecies (*A. o. oratrix*) and geographic source population (coastal eastern Mexico) of smuggled parrots. As laboratory protocols and bioinformatic pipelines for degraded DNA continue to improve, museum collections will become even more powerful tools in conservation genomics, providing critical baselines for monitoring genetic diversity, identifying poaching hotspots, and supporting forensic investigations of illegal wildlife trade and crimes against wildlife.

### 4.3 Taxonomic Implications for Amazona oratrix

Although clarifying the evolutionary history of *A. oratrix* was not the primary goal of this study, our findings offer meaningful taxonomic insights that validate results from a recent DNA study (Escalante‐Vargas and Escalante‐Pliego) with genome‐scale data. We recovered a clear genetic division between *A. o. oratrix* populations on the Pacific and Atlantic slopes of Mexico (F_ST_ = 0.25; Figure 2), supporting the resurrection of the subspecies *A. o. magna* for birds inhabiting the Atlantic coastal lowlands of eastern Mexico (Monroe and Howell 1966). For the subspecies *A. o. oratrix*, the type locality of Petapa, Oaxaca, lies geographically close to our sampling in Oaxaca (population 4; Figure 2A), implying that the type specimen belongs to this Pacific coastal clade, and therefore that this clade should retain the moniker *A. o. oratrix*. Additionally, our results affirm the distinctiveness of the Belize population, which forms a separate genetic cluster supporting the continued recognition of *A. o. belizensis* as a valid subspecies.

The genomic ancestry of a single specimen of the closely related Yellow‐naped Parrot ( *A. auropalliata* ) from Chiapas fell between the three major *A. oratrix* clusters in PCA space and in a phylogenetic network. This pattern is consistent with prior phylogenetic studies suggesting that *A. auropalliata* is nested within *A. oratrix* and is more appropriately treated as a subspecies of *A. oratrix* (Eberhard and Bermingham 2004; Escalante‐Vargas and Escalante‐Pliego 2025; Ribas et al. 2007). Future studies should undertake comprehensive genomic sampling across the full range of the *Amazona ochrocephala* complex ( *A. oratrix* + *A. auropalliata* + *A. ochrocephala* ), a group that has long been recognized as taxonomically challenging (Eberhard and Bermingham 2004; Ribas et al. 2007). Another area of remaining taxonomic uncertainty is the subspecies *A. o. hondurensis*. The single individual that we sequenced from this subspecies failed genomic filtering protocols and could not be included in the analyses presented in this manuscript. Future genomic work incorporating genomic sampling from this population will be essential for fully resolving the systematics of the *A. oratrix/auropalliata* complex.

## 5 Conclusions: Conservation and Policy Implications

This study demonstrates the power of mining historical museum specimens for genomic data to support wildlife conservation, enforcement, and policy. By identifying the likely origin of smuggled *A. oratrix* nestlings to southeastern Mexico, we provide actionable information for repatriation efforts and for decisions about which geographic regions ought to be targeted in conservation efforts. Southeastern Mexico has been previously recognized as a hotspot for parrot smuggling, as Veracruz, Campeche, and Tabasco have three of the seven highest seizures of parrots by state in all of Mexico (Saldaña et al. 2007). Future efforts to link point of seizure to source populations will further elucidate trade routes of poached parrots.

Genetic assignment methods like the ones we employ here can also inform decisions about the repatriation of confiscated individuals, helping avoid unintentional genetic mixing that could compromise local adaptation via outbreeding (Gentile et al. 2013). More broadly, our results show how museum collections, long curated for basic research, can also serve as reference libraries for forensic applications, enabling rapid response to ongoing trafficking threats. As genomic sequencing has become affordable and commonplace, it has become one of the most powerful tools in the toolboxes of biologists interested in conservation and wildlife forensics. Yet, this genomic approach is inherently limited by the availability of genetic samples from across a species' range and can only be truly utilized to its full potential when the genetic diversity of a species can be surveyed in a comprehensive geographic context. Here we've demonstrated that historical museum specimens sourced from natural history museum collections have the potential to fill this crucial sampling gap and facilitate our ability to track and even repatriate trafficked animals across the globe.

## Funding

This work was supported by the Harold and Colene Brown Family Foundation and the National Science Foundation (DEB‐1652979).

## Conflicts of Interest

The authors declare no conflicts of interest.

## Supporting information


**Table S1:** Genomic sequencing table. “sample_ID” corresponds to the unique ID of each sample. “Collection” refers to the museum collection that each sample was sourced from. MLZ = Moore Laboratory of Zoology; UMMZ = University of Michigan Museum of Zoology; ANSP = Academy of Natural Sciences, Philadelphia; LSUMZ = Louisiana State University Museum of Natural Science; BBR = Belize Bird Rescue. “Sampling locality” refers to the corresponding numbered locality on the sampling map. “Sample type” refers to the source material used for DNA extraction from each sample. “passed genomic filtering?” states whether the given sample was removed during the filtering steps described in the methods section. Samples with “Y” in this column were used in downstream analyses. “proportion missing data” reports the proportion of missing genotypes for each sample, among the 2,274 SNPs in the filtered SNP dataset. “Mean genotype depth” reports the mean number of sequencing reads across the called genotypes in the filtered SNP dataset, for a given sample.
**Table S2:** Final SNP counts for each Locator prediction run.
**Table S3:** Summary statistics for Locator model performance for each subset of *A. oratrix* locality predictions. Model performance was evaluated for each run of the models using coefficients of determination (*R* ^2^) for latitude and longitude, as well as mean and median prediction error (in degrees). Representative models with mean error values closest to the average across the runs are highlighted in gray and used for Figure 2 and S1.
**Figure S1:** Representative run of Locator predictions for the known Tamaulipas sample (ao_MLZ_48333) with calibration points from all 12 populations. The black point represents the mean of the 200 bootstrap predictions, which was ~361 km away from the true locality of the sample (site 7). Individuals are color‐coded and labeled according to the sampling locality in Figure 1A.
**Figure S2:** Principal Component Analysis from 1598 unlinked SNPs shows similar sample clustering patterns as the PCA conducted with the entire 2274 SNP dataset (Figure 2B).

## Data Availability

The code and SNP datasets that support the findings of this study are hosted in the following GitHub repository: https://github.com/brendarramirez/yellow_headed_parrot. The raw sequence data for all 54 Yellow‐headed Parrot samples are publicly available from NCBI's Sequence Read Archive, via BioProject PRJNA1475542 (https://www.ncbi.nlm.nih.gov/bioproject/PRJNA1475542).
