# Supplementary material for: Unlocking the Past to Protect the Future: Forensic DNA and Historical Specimens Reveal the Origins of Smuggled Yellow‐Headed Parrots
Source: Evol Appl. 2026 Jun 14;19(6):e70282. doi: 10.1111/eva.70282 (PMC13266085; doi:10.1111/eva.70282)
Supplement: Supplementary file 1 — Table S1: Genomic sequencing table. “sample_ID” corresponds to the unique ID of each sample. “Collection” refers to the museum collection that each sample was sourced from. MLZ = Moore Laboratory of Zoology; UMMZ = University of Michigan Museum of Zoology; ANSP = Academy of Natural Sciences, Philadelphia; LSUMZ = Louisiana State University Museum of Natural Science; BBR = Belize Bird Rescue. “Sampling locality” refers to the corresponding numbered locality on the sampling map. “Sample type” refers to the source material used for DNA extraction from each sample. “passed genomic filtering?” states whether the given sample was removed during the filtering steps described in the methods section. Samples with “Y” in this column were used in downstream analyses. “proportion missing data” reports the proportion of missing genotypes for each sample, among the 2,274 SNPs in the filtered SNP dataset. “Mean genotype depth” reports the mean number of sequencing reads across the called genotypes in the filtered SNP dataset, for a given sample. Table S2: Final SNP counts for each Locator prediction run. Table S3: Summary statistics for Locator model performance for each subset of A. oratrix locality predictions. Model performance was evaluated for each run of the models using coefficients of determination (R 2) for latitude and longitude, as well as mean and median prediction error (in degrees). Representative models with mean error values closest to the average across the runs are highlighted in gray and used for Figure 2 and S1. Figure S1: Representative run of Locator predictions for the known Tamaulipas sample (ao_MLZ_48333) with calibration points from all 12 populations. The black point represents the mean of the 200 bootstrap predictions, which was ~361 km away from the true locality of the sample (site 7). Individuals are color‐coded and labeled according to the sampling locality in Figure 1A. Figure S2: Principal Component Analysis from 1598 unlinked SNPs shows similar s [file EVA-19-e70282-s001.docx]

**Table S1**. Genomic sequencing table. ‘sample_ID’ corresponds to the unique ID of each sample. ‘Collection’ refers to the museum collection that each sample was sourced from. MLZ = Moore Laboratory of Zoology; UMMZ = University of Michigan Museum of Zoology; ANSP = Academy of Natural Sciences, Philadelphia; LSUMZ = Louisiana State University Museum of Natural Science; BBR = Belize Bird Rescue. ‘Sampling locality’ refers to the corresponding numbered locality on the sampling map. ‘Sample type’ refers to the source material used for DNA extraction from each sample. ‘passed genomic filtering?’ states whether the given sample was removed during the filtering steps described in the methods section. Samples with ‘Y in this column were used in downstream analyses. ‘proportion missing data’ reports the proportion of missing genotypes for each sample, among the 2,274 SNPs in the filtered SNP dataset. ‘Mean genotype depth’ reports the mean number of sequencing reads across the called genotypes in the filtered SNP dataset, for a given sample.

| **sample_ID** | **Collection** | **Catalog number** | **Species** | **Subspecies** | **Sampling locality** | **State, Country** | **Latitude** | **Longitude** | **sample type** | **passed genomic filtering?** | **proportion missing data** | **mean genotype depth** |
| --- | --- | --- | --- | --- | --- | --- | --- | --- | --- | --- | --- | --- |
| ao_ANSP_90568 | ANSP | 90568 | *oratrix* | *hondurensis* | - | Yoro, Honduras | 15.62 | -87.79 | toepad | N |  |  |
| ao_LSUMZ_23890 | LSUMZ | 23890 | *oratrix* | *oratrix* (east) | - | Tabasco, Mexico | 17.94 | -91.53 | toepad | N |  |  |
| ao_LSUMZ_33050 | LSUMZ | 33050 | *oratrix* | *oratrix* (west) | 4 | Oaxaca, Mexico | 16.39 | -98.37 | toepad | Y | 0.02 | 73.96 |
| ao_LSUMZ_39731 | LSUMZ | 39731 | *auropalliata* | *auropalliata* | 5 | Chiapas, Mexico | 16.13 | -93.72 | toepad | Y | 0.02 | 55.02 |
| ao_LSUMZ_43831 | LSUMZ | 43831 | *oratrix* | *oratrix* (west) | 2 | Colima, Mexico | 19.09 | -104.1 | toepad | Y | 0.16 | 40.15 |
| ao_LSUMZ_43832 | LSUMZ | 43832 | *oratrix* | *oratrix* (west) | 2 | Colima, Mexico | 19.09 | -104.1 | toepad | Y | 0.17 | 24.49 |
| ao_MLZ_32244 | MLZ | 32244 | *oratrix* | *oratrix* (east) | - | Tamaulipas, Mexico | 22.8 | -98.71 | toepad | N |  |  |
| ao_MLZ_35920 | MLZ | 35920 | *oratrix* | *oratrix* (east) | 9 | Veracruz, Mexico | 19.24 | -96.38 | toepad | Y | 0.54 | 9.59 |
| ao_MLZ_39530 | MLZ | 39530 | *oratrix* | *oratrix* (east) | - | Tamaulipas, Mexico | 23.32 | -99.02 | toepad | N |  |  |
| ao_MLZ_40633 | MLZ | 40633 | *oratrix* | *oratrix* (east) | - | Tamaulipas, Mexico | 23.97 | -99.11 | toepad | N |  |  |
| ao_MLZ_40634 | MLZ | 40634 | *oratrix* | *oratrix* (east) | 6 | Tamaulipas, Mexico | 23.97 | -99.11 | toepad | Y | 0.03 | 24.39 |
| ao_MLZ_40635 | MLZ | 40635 | *oratrix* | *oratrix* (east) | - | Tamaulipas, Mexico | 23.97 | -99.11 | toepad | N |  |  |
| ao_MLZ_41497 | MLZ | 41497 | *oratrix* | *oratrix* (east) | 8 | San Luis Potosí, Mexico | 22.37 | -99.29 | toepad | Y | 0.21 | 14.92 |
| ao_MLZ_45517 | MLZ | 45517 | *oratrix* | *oratrix* (west) | - | Oaxaca, Mexico | 16.5 | -94.43 | toepad | N |  |  |
| ao_MLZ_48333 | MLZ | 48333 | *oratrix* | *oratrix* (east) | 7 | Tamaulipas, Mexico | 23.2 | -98.44 | toepad | Y | 0.25 | 15.46 |
| ao_MLZ_50773 | MLZ | 50773 | *oratrix* | *oratrix* (west) | 4 | Guerrero, Mexico | 16.47 | -98.41 | toepad | Y | 0.31 | 14.19 |
| ao_MLZ_50774 | MLZ | 50774 | *oratrix* | *oratrix* (west) | 4 | Guerrero, Mexico | 16.47 | -98.41 | toepad | Y | 0.24 | 20.02 |
| ao_MLZ_50775 | MLZ | 50775 | *oratrix* | *oratrix* (west) | 4 | Guerrero, Mexico | 16.47 | -98.41 | toepad | Y | 0.03 | 32.66 |
| ao_MLZ_59507 | MLZ | 59507 | *oratrix* | *oratrix* (east) | 10 | Veracruz, Mexico | 17.562 | -95.107 | toepad | Y | 0.47 | 12.89 |
| ao_UMMZ_103984 | UMMZ | 103984 | *oratrix* | *oratrix* (east) | 11 | Tabasco, Mexico | 17.8 | -91.53 | toepad | Y | 0.01 | 93.95 |
| ao_UMMZ_130517 | UMMZ | 130517 | *oratrix* | *oratrix* (west) | 3 | Michoacán, Mexico | 18.53 | -103.59 | toepad | Y | 0.03 | 31.89 |
| ao_UMMZ_95618 | UMMZ | 95618 | *oratrix* | *tresmariae* | 1 | Nayarit, Mexico | 21.4538685 | -106.413795 | toepad | Y | 0.01 | 65.93 |
| ao_UMMZ_95619 | UMMZ | 95619 | *oratrix* | *tresmariae* | 1 | Nayarit, Mexico | 21.4538685 | -106.413795 | toepad | Y | 0.01 | 93.61 |
| ao_MLZ_70063 | MLZ | 70063 | *oratrix* | *unknown* | 13 | California, USA | 34.1478 | -118.1445 | tissue | Y | 0 | 95.51 |
| ao_MLZ_70074 | MLZ | 70074 | *oratrix* | *unknown* | 13 | California, USA | 34.184714 | -118.147949 | tissue | Y | 0 | 199.38 |
| ao_MLZ_1105 | MLZ | 70287 | *oratrix* | *unknown* | 14 | California, USA | 33.196333 | -116.799171 | tissue | Y | 0 | 127.82 |
| ao_BC_107 | - | - | *oratrix* | *belizensis* | 12 | Belize | 17.319 | -88.553 | blood | Y | 0.03 | 18.82 |
| ao_BC_108 | - | - | *oratrix* | *belizensis* | 12 | Belize | 17.319 | -88.553 | blood | Y | 0.19 | 9.72 |
| ao_BC_109 | - | - | *oratrix* | *belizensis* | 12 | Belize | 17.319 | -88.553 | blood | Y | 0.18 | 10.31 |
| ao_BC_A112 | - | - | *oratrix* | *belizensis* | - | Belize | 17.319 | -88.553 | blood | N |  |  |
| ao_BC_A113 | - | - | *oratrix* | *belizensis* | 12 | Belize | 17.319 | -88.553 | blood | Y | 0.1 | 11.77 |
| ao_BC_A114 | - | - | *oratrix* | *belizensis* | 12 | Belize | 17.319 | -88.553 | blood | Y | 0.05 | 16.7 |
| ao_BC_A115 | - | - | *oratrix* | *belizensis* | 12 | Belize | 17.319 | -88.553 | blood | Y | 0.03 | 19.2 |
| ao_BC_A116 | - | - | *oratrix* | *belizensis* | 12 | Belize | 17.319 | -88.553 | blood | Y | 0.09 | 11.39 |
| ao_BC_A117 | - | - | *oratrix* | *belizensis* | 12 | Belize | 17.319 | -88.553 | blood | Y | 0.16 | 9.18 |
| ao_BC_A118 | - | - | *oratrix* | *belizensis* | 12 | Belize | 17.319 | -88.553 | blood | Y | 0.04 | 16.88 |
| ao_SP_1 | - | - | *oratrix* | unknown | - | unknown | - | - | blood | Y | 0.03 | 20.23 |
| ao_SP_2 | - | - | *oratrix* | unknown | - | unknown | - | - | blood | Y | 0.02 | 24.49 |
| ao_SP_3 | - | - | *oratrix* | unknown | - | unknown | - | - | blood | Y | 0.01 | 25.92 |
| ao_SP_4 | - | - | *oratrix* | unknown | - | unknown | - | - | blood | Y | 0.01 | 33.59 |
| ao_SP_5 | - | - | *oratrix* | unknown | - | unknown | - | - | blood | Y | 0.02 | 21.77 |
| ao_SP_6 | - | - | *oratrix* | unknown | - | unknown | - | - | blood | Y | 0.01 | 24.91 |
| ao_SP_7 | - | - | *oratrix* | unknown | - | unknown | - | - | blood | Y | 0 | 41.71 |
| ao_SP_8 | - | - | *oratrix* | unknown | - | unknown | - | - | blood | Y | 0.01 | 31.19 |
| ao_SP_831 | - | - | *oratrix* | unknown | - | unknown | - | - | blood | Y | 0 | 44.87 |
| ao_SP_832 | - | - | *oratrix* | unknown | - | unknown | - | - | blood | Y | 0.01 | 40.39 |
| ao_SP_833 | - | - | *oratrix* | unknown | - | unknown | - | - | blood | Y | 0.01 | 39.67 |
| ao_SP_834 | - | - | *oratrix* | unknown | - | unknown | - | - | blood | Y | 0 | 44.63 |
| ao_SP_835 | - | - | *oratrix* | unknown | - | unknown | - | - | blood | Y | 0 | 43.3 |
| ao_SP_836 | - | - | *oratrix* | unknown | - | unknown | - | - | blood | Y | 0 | 47.91 |
| ao_SP_837 | - | - | *oratrix* | unknown | - | unknown | - | - | blood | Y | 0 | 57.02 |
| ao_SP_838 | - | - | *oratrix* | unknown | - | unknown | - | - | blood | Y | 0 | 38.38 |
| ao_SP_839 | - | - | *oratrix* | unknown | - | unknown | - | - | blood | Y | 0.01 | 35.2 |
| ao_SP_840 | - | - | *oratrix* | unknown | - | unknown | - | - | blood | Y | 0 | 47.41 |

**Table S2.** Final SNP counts for each Locator prediction run.

| Locator Prediction Run | Number of Samples | Number of SNPs |
| --- | --- | --- |
| ao_MLZ_48333 | 18 | 1,312 |
| Confiscated YHPA | 36 | 1,972 |
| Rescue YHPA | 21 | 1,464 |

**Table S3**. Summary statistics for Locator model performance for each subset of *A. oratrix* locality predictions. Model performance was evaluated for each run of the models using coefficients of determination (R^2^) for latitude and longitude, as well as mean and median prediction error (in degrees). Representative models with mean error values closest to the average across the runs are highlighted in gray and used for Figure 2 and S1.

**Tamaulipas (ao_MLZ_48333) Predictions**

| Set Seed | R^2^(x) | R^2^(y) | Mean Error | Median Error |
| --- | --- | --- | --- | --- |
| 835 | 0.989 | 0.982 | 3.847 | 5.086 |
| 163 | 0.634 | 0.765 | 4.843 | 4.635 |
| 321 | 0.996 | 0.986 | 2.806 | 3.381 |
| 958 | 0.979 | 0.998 | 4.249 | 4.669 |
| 841 | 0.563 | 0.86 | 2.662 | 2.13 |
| Averages | 0.832 | 0.918 | 3.681 | 3.98 |

**Confiscated YHPA Predictions**

| Set Seed | R^2^(x) | R^2^(y) | Mean Error | Median Error |
| --- | --- | --- | --- | --- |
| 835 | 0.937 | 0.859 | 1.991 | 1.112 |
| 163 | 0.944 | 0.098 | 4.641 | 5.44 |
| 321 | 0.967 | 0.046 | 3.354 | 1.534 |
| 958 | 0.088 | 0.997 | 3.18 | 2.994 |
| 841 | 0.324 | 0.894 | 3.827 | 2.752 |
| Averages | 0.652 | 0.579 | 3.399 | 2.766 |

**Rescue YHPA Predictions**

| Set Seed | R^2^(x) | R^2^(y) | Mean Error | Median Error |
| --- | --- | --- | --- | --- |
| 835 | 0.674 | 0.884 | 2.473 | 2.245 |
| 163 | 0.695 | 0.386 | 6.494 | 6.394 |
| 321 | 0.994 | 0.952 | 3.545 | 3.792 |
| 958 | 0.860 | 0.968 | 2.891 | 2.589 |
| 841 | 0.860 | 0.965 | 2.314 | 2.097 |
| Averages | 0.817 | 0.831 | 3.543 | 3.423 |
|  |  |  |  |  |


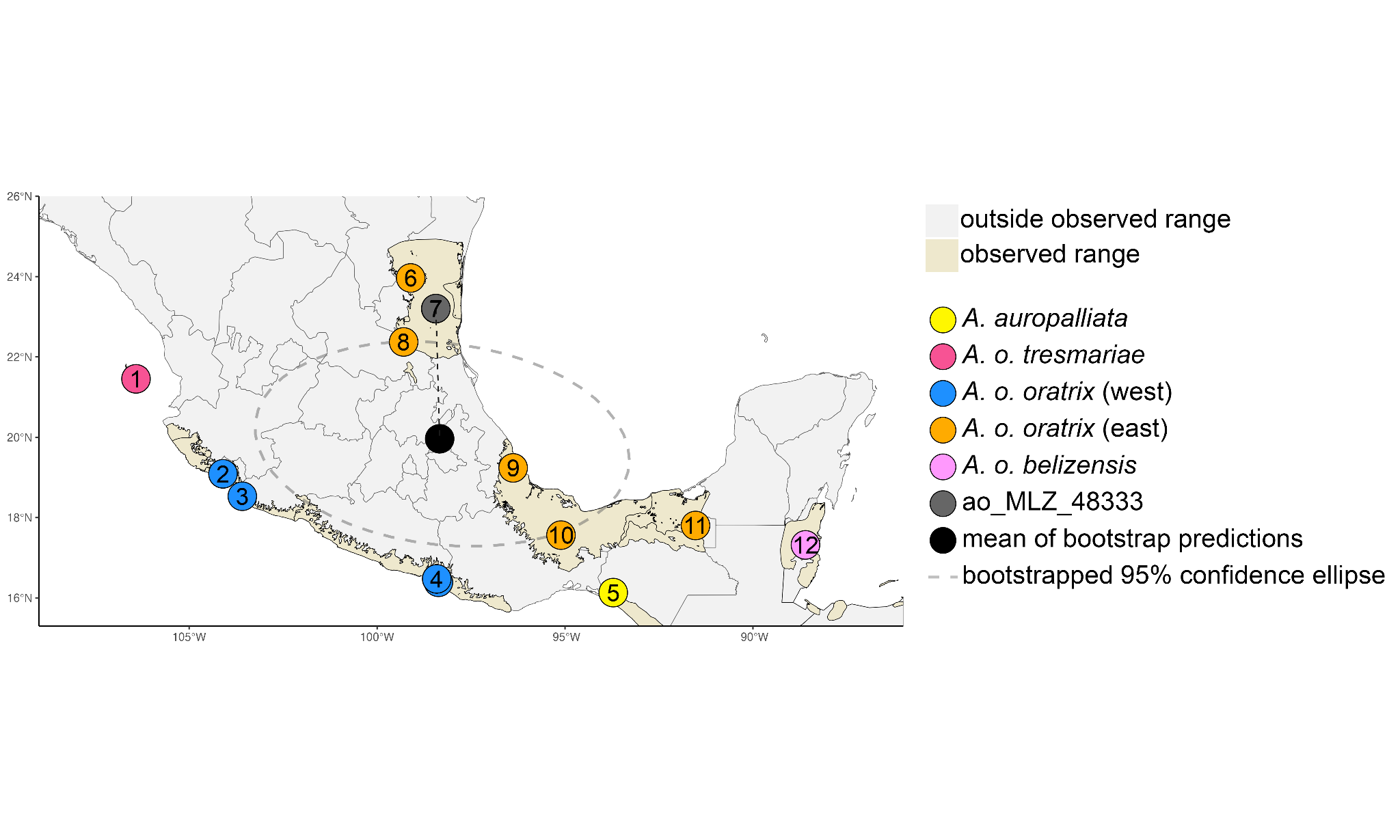


**Figure S1**. Representative run of Locator predictions for the known Tamaulipas sample (ao_MLZ_48333) with calibration points from all 12 populations. The black point represents the mean of the 200 bootstrap predictions, which was ~361km away from the true locality of the sample (site 7). Individuals are color-coded and labeled according to the sampling locality in Figure 1A.


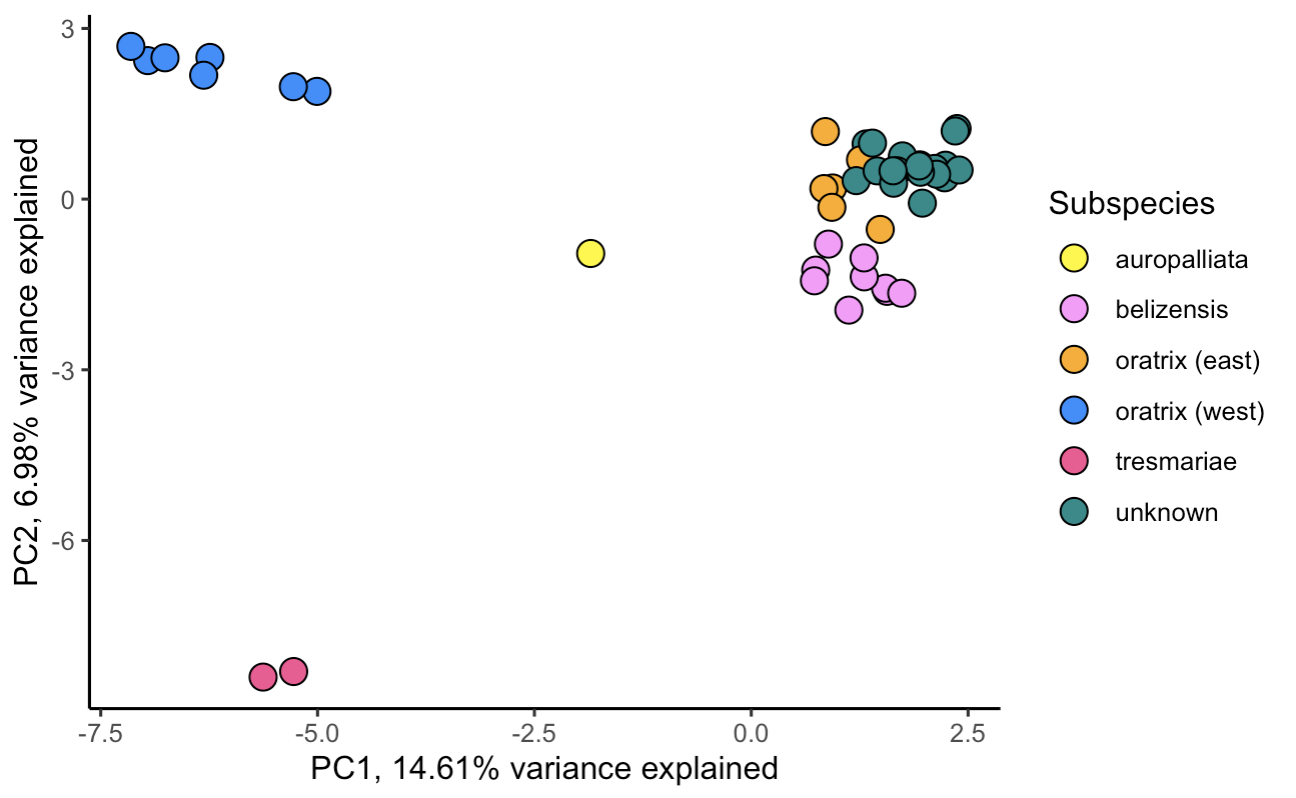


**Figure S2.** Principal Component Analysis from 1,598 unlinked SNPs shows similar sample clustering patterns as the PCA conducted with the entire 2,274 SNP dataset (Fig. 2B).
